# Evaluating Implementation of the Behavioral Recovery Outreach (BRO) Team: One Year of Implementation

**DOI:** 10.1093/geroni/igab046.173

**Published:** 2021-12-17

**Authors:** Jenefer Jedele, Cameron Griffin, Kathleen Matthews, Latrice Vinson

**Affiliations:** 1 Veterans Health Administration, Ann Arbor, Michigan, United States; 2 VA, Ann Arbor, Michigan, United States; 3 VISN 23, Des Moines, Iowa, United States; 4 Department of Veterans Affairs, Department of Veterans Affairs, District of Columbia, United States

## Abstract

We present evaluation results after one year of implementation by nine BRO Teams. Monthly checklists documented consistent composition across teams: a psychologist, social worker and nurse. Social workers were recognized as having a critical role in implementation, serving as a referral source and liaison between the CLC, Veteran/family, and community facility. Early implementation focused on team and program development with barriers including unprotected time for Team members. In the first year, the nine teams enrolled 70 Veterans, discharging 86% to community facilities. Characteristics of the Veterans suggest Teams are reaching the complex Veteran targeted by the model. Barriers to successful discharge include community facility inexperience/training and confidence to manage complex residents. COVID emerged as the leading barrier to outreach to internal and external partners and providing transitional support to the Veteran after discharge. We discuss the impact of these preliminary findings on future implementation and dissemination of the model.

